# Fat

**Published:** 1889-02-09

**Authors:** 


### Physiology and its Uses.

(*Continued from p.* 167.)

### FAT.

The subject of "fat" is one in which all men and women who are approaching middle age take a deep and peculiar interest. Some people are "fat" even as boys and girls; but the majority are not so. When the man whose domestic register informs him that the age of thirty-five has certainly been passed, begins to notice an unmistakable tightening about the waistband, and an equally demonstrable prominence and rotundity below that level, he feels that the period of youth, with all its delights, all its careless freedom, and all its remoteness from old age, has at last gone. "Gone, gone, never more to return." Various are the devices resorted to for staying or hiding the approach of the good-natured but resolute enemy, obesity. The soldier straps himself with a savage tightness, as if he would say, "I will not be fat." The advancing matron, whose head and heart are still seventeen although her figure is seven-and-thirty, has methods known only to herself and certain artistes in the corset line. The untaxed bachelor of forty-five, whose clean-shaven face and faultless frock-coat try to persuade him that he is still five-and-twenty, looks down with speechless horror when he finds his feet beginning to be hidden by a hemispherical prominence below the waistcoat, which proclaims, as with a bellman's sounding howl, that "forty" belongs to the dim and distant past. Alas! that so jolly and prosperous a fellow as "Fat" should be the herald to announce that youth is finally gone, and that middle age, with all its deprivations, its tame joys and its sober prospects, has definitely arrived.

But "Fat" is really a friend, although he is treated so discourteously. Moreover, in our judgment, he does not spoil the outward appearance, at least in persons who take plenty of exercise and maintain robust health. No doubt if the middle-aged man becomes lazy, and neglects his daily walk, or tricycle ride, or wood-chopping, or what not, the fat will accumulate in disproportionate quantities, and give him the appearance of a woolsack on legs. But that is, as a rule, entirely the fat man's own fault; if he will be lazy, he must pay the penalty. "Fat" is not a very "profound" subject, and we may be excused if we give the main points about it in a discursive rather than in a severely scientific form. To begin with, it consists of small cells or vesicles, which usually vary in size from the 300th to the 600th of an inch in diameter. The natural shape of a fat vesicle is round or oval, but when the cells are squeezed together, as invariably happens in regions where the fat accumulates, the shapes will vary markedly from the normal. Each separate vesicle or cell is a thin envelope filled with oily matter, at the ordinary temperature of the body. There is nothing easier to detect under the microscope than a fat cell. It usually looks as rotund and good-natured as a fat man who likes being fat. Of course, when the body cools in death, the fat assumes the hard, firm appearance which is so familiar in the fat of animals.

"Adipose tissue," which is the learned name for fat, is distributed over almost the whole of the body. It is found, for example, as a "subcutaneous layer" under the greater part of the skin. Beneath some portions, and in certain persons, it is enormously thick. In this situation it is held in place by what is called "areolar" or netted "tissue," the fat and its containing tissue constituting the *panniculus adiposus.* Certain internal parts are often completely embedded in fat; the kidney, for example, as everyone may know who has ever carved, or seen carved, a loin of mutton or pork. Collections of fat are common round the joints, and indeed in almost every part where "padding" is required. The "marrow" of the bones consists very largely of fat, and it is of such a kind that epicures consider it one of the choicest morsels known to gastronomic art. There are a few parts of the body in which fat is unknown: for example, there is none inside the cavity of the skull, or under the eyelids, or in the lungs. Almost everywhere else fat may show itself, and if not in excess may serve useful and important purposes.

The common fat of the human body is said to consist almost entirely of two substances—"margarin," which is solid and fatty, and "olein," which is fluid and oily. Therein it differs from the fat of other animals, such as the cow and the sheep. The fatty parts of those animals consist of "stearin," which is firm, and olein, which is oily and fluid. Stearin and margarin are not, however, very dissimilar, and belong to the same class of substances, "Oleo-margarine," if pure and worthy of the name, should be very suitable as a substitute for butter, because of its kinship with the normal fat of the human body. Margarin consists of glycerine as a base, in combination with margaric acid. Similarly, glycerine constitutes the base of stearin, but the acid with which it is combined in this case is, of course, "stearic" acid. The fatty parts of the body are well supplied with bloodvessels, showing that Nature has a very definite purpose to keep up the supply. Fat has no nerves, except that sometimes a nerve-trunk will pass through fatty structures on its way to its final distribution. But it has no nerves of its own, and therefore does not "feel."

What are the chief uses of fat? In moderate quantities and suitable localities it undoubtedly "pads," "rounds up," and improves the figure. That, however, does not perhaps deserve to be called a primary use. There are two purposes which it serves, and which are of great importance in the animal economy. It constitutes a reserve store of nutrition for the body, and it checks the radiation of heat. These are two functions very necessary, and indeed essential, to perfect health. In ultimate analysis fat consists chiefly of carbon and hydrogen. It is readily absorbed by the blood, and consumed in the process of respiration; thereby contributing to the generation of heat in the body. It is believed that when too much carbon and hydrogen are introduced into the blood by the digestive processes, the excess, which is not at the moment required, is stored up in the fat cells of the economy; and that, conversely, when too little carbon and hydrogen are extracted from the food by digestion, these elements, stored up in the fat, are given out to supply the deficiency. Fat may therefore be looked upon as accumulated funds, and in moderate quantities is a distinct gain. In excess it is like any other plethora, and does more harm than good. Hybernating animals, such as the hedgehog, well illustrate the use of fat as a stored nutrient. They sleep all winter, and appear to live upon nothing else but their accumulated fat. Of course life is carried on at the lowest possible level; it is existence merely. Nevertheless, the return of spring is to them a new resurrection, and the question is, whether their long sleep is not the best possible way of passing the long, cold, and hungry season of winter.

It is well known that fat is a bad conductor of heat. It ought therefore to be a protection against the sun in summer, keeping his rays out, as well as against the frost in winter, keeping the warmth in. These seem rather contradictory functions for one and the same substance to perform; but it is probable that they are quite reconcilable. If it be argued that a fat man is sooner hot in summer than a lean one, the reason is, not that the heat of the sun raises his temperature more quickly, but that his forced and struggling movements cause a great production of internal heat, which is prevented from radiation by the all-enclosing case of fat which surrounds him. But whatever be the practical demerits of fat in summer, in winter the advantages are all the other way. In warm-blooded creatures, especially those exposed to severe cold, such as the whale and other cetaceans, the fat forms an exceedingly thick stratum underneath the skin, and, as an eminent anatomist has said, "must prove a much more effectual protection than a covering of fur in the watery element." It has been estimated that ordinarily about a twentieth part of the weight of the human body consists of fat. Be that as it may, it is clear that fat is essential to health, and that in proper quantities it not only does not diminish, but adds to the comeliness both of men and women.

